# Eco-Friendly Species *Evernia prunastri* (L.) Ach.: Phenolic Profile, Antioxidant, Anti-inflammatory, and Anticancer Properties

**DOI:** 10.1021/acsomega.3c10407

**Published:** 2024-11-05

**Authors:** Khadidja Belhouala, Cansu Korkmaz, Meltem Taş Küçükaydın, Selçuk Küçükaydın, Mehmet Emin Duru, Bachir Benarba

**Affiliations:** † Laboratory Research on Biological Systems and Geomatics, Mustapha Stambouli University of Mascara, Mascara 29001, Algeria; ‡ Department of Biology, Faculty of Science, Muğla Sıtkı Koçman University, 48000 Muğla, Turkey; § Department of Chemistry, Faculty of Science, Muğla Sıtkı Koçman University, 48000 Muğla, Turkey; ∥ Department of Medical Services and Techniques, Köyceğiz Vocational School of Health Services, Muğla Sıtkı Koçman University, 48800 Köyceğiz/Muğla, Turkey; ⊥ Thematic Research Agency in Health and Life Sciences (ATRSSV), 31000 Oran, Algeria

## Abstract

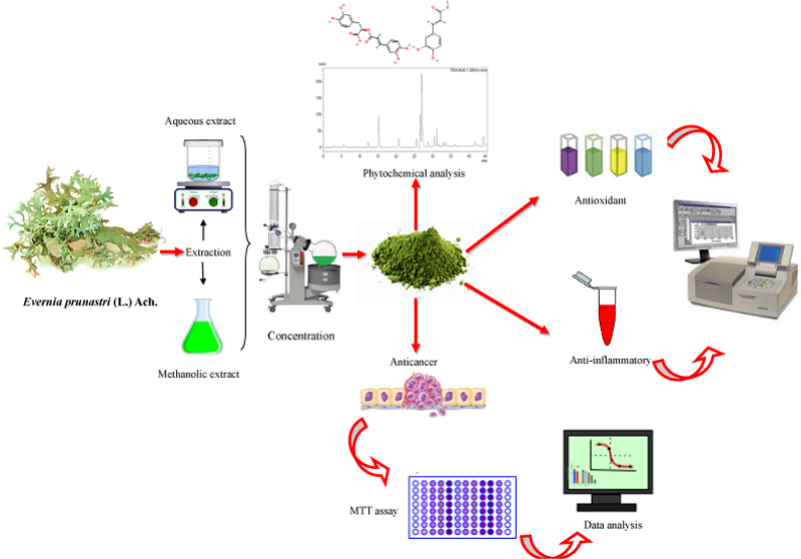

Growing in prominence is the utilization of natural product-based therapies, especially edibles used in traditional medicine, as more people seek natural and holistic approaches to health and well-being. This research aimed to determine the phenolic compounds, and antioxidant, anti-inflammatory, and anticancer effects of aqueous and methanolic extracts from *Evernia prunastri* (L.) Ach., a common spice in Algeria. HPLC-DAD was used to establish the phenolic profile, whereas the antioxidant activity was determined by DPPH, FRAP, phosphomolybdate, and hydrogen peroxide (H_2_O_2_) assays. Human red blood cell (HRBC) stabilization, albumin denaturation, and proteinase inhibition procedures were performed to investigate the anti-inflammatory activities, and an MTT assay was used to demonstrate the cytotoxic effect against three human cancer cell lines (HT-29, PC-3, A549) and human nontumor (CCD18-Co) cells. Our results showed that the major phenolics detected were mostly phenylpropanoids with domination of rosmarinic acid (79.17 mg/g), caffeic acid (46.52 mg/g), *trans*-cinnamic acid (29.23 mg/g), and chlorogenic acid (23.68 mg/g). In addition, six flavonoids were identified (1.98–11.34 mg/g), namely, luteolin, myricetin, kaempferol, rutin, apigenin, and quercetin. Other compounds were relatively present in both extracts, gallic acid and 3-hydroxybenzoic acid (phenolic acids), except pyrocatechol (benzenediol), which was slightly detected in the aqueous extract (0.91 mg/g). Moreover, both *E. prunastri* extracts showed strong antioxidant activity, with a higher antioxidant potential, as shown by the methanolic extract. Likewise, both reduced HRBC hemolysis damage and moderately suppressed protein denaturation, which reflected their anti-inflammatory potential. Interestingly, the methanolic extract significantly reduced the growth of HT-29, PC-3, and A549 cells by 67.03, 75.56, and 62.96% respectively. No cytotoxic effects were observed in the nontumor cells. The methanolic extract had the lowest IC_50_ values of 100 ± 0.04, 146 ± 0.05, and 112 ± 0.06 μg/mL against HT-29, PC-3, and A549 cell lines, respectively. In conclusion, *E. prunastri*, especially its methanolic extract, could be considered as a promising source of antioxidant and anticancer molecules.

## 1 Introduction

Lichens (lichen-forming fungi) represent the dominant vegetation of approximately 8% of terrestrial ecosystems with about one-fifth of the total known fungal species until now.^1,2^ In fact, over 20,000 known species of lichens have already been described around the world spreading in diverse ecosystems from arctic tundra to desert climates.^3^ These species are basically symbiotic organisms composed of fungus and photosynthetic (algal or cyanobacterial) partners growing on barks, stems, leaves, and in soil and mostly in environments that are not as conducive to higher plant life.^4,5^

As one of the largest families of lichenized Ascomycota, Parmeliaceae consists of 2700 species (10% of the total number of lichen species). For the most part, some of them have long been used as spices in foods, as colorants in the production of alcohol and perfumes, and as traditional treatments in folk medicine for many infectious diseases.^6,7^ Owing to their pharmacological properties including analgesic, antimicrobial, antioxidant, antipyretic, antimycotic, anti-inflammatory, antiproliferative, antiviral, and cytotoxic effects,^8−14^ these organisms revealed important secondary metabolite classes (depsides, depsidones, dibenzofurans, xanthones, terpene derivatives, etc.), beside enzymes, polysaccharides, and fatty acids.^15−17^

Most lichens play an important role of phenols, including depsides, depsidones, and dibenzofurans, in the antioxidant activity because of their ability to scavenge free radicals.^9^ Of this lichen family, *Evernia prunastri* (L.) Ach., a common epiphytic lichen, coexists in many mountainous temperate forests of the Northern Hemisphere, favoring oaks and pines.^18,19^*E. prunastri*, also known as “oakmoss”, was reported to be used against cancer, epilepsy, and gastrointestinal diseases in Algeria.^20,21^ Previously, a few studies have focused on the evaluation of secondary metabolites of lichen (atranorin), which may be responsible for the curative potential, as well as other valuable substances for which information is limited in this regard. *E. prunastri* extracts were tested active against Gram-positive (*Bacillus subtilis* and *Staphylococcus aureus*) bacterial strains in a previous report.^22^ Some anticancer properties have also been featured for *E. prunastri* extracts due to the presence of usnic acid.^23^ Moreover, cytotoxic effects against mouse melanoma and rat glioma cell lines have been reported.^24^ The antioxidant and anti-inflammatory activities of β-glucan-containing polysaccharide extracts of this lichen species and their effects on some cancer cells have been reported. However, in this study, *β-*glucan-rich extracts obtained by different extractions were taken into consideration.^24^ When the literature is examined, it is understood that various biological activity studies have been reported on *E. prunastri* growing under different ecological conditions. However, no study has been observed on this lichen, which naturally grows on oak trees in the mountainous climate of Algeria and is traditionally used among the local people.^20^ Although studies have been conducted on Moroccan *E. prunastri*, they have been limited to HeLa cancer cells with antifungal, antibacterial, and radical scavenging activity.^11^

With the increasing incidence of deadliest diseases such as cancers and adverse side effects with the rising cost of chemical drugs, there is an increasing reliance on natural products. The search for drugs from natural sources using computational methods has led to the discovery of important, effective, and safe molecules and the development of other nutraceuticals.^25−27^ In drug development, in silico approaches including ADMET and molecular docking analysis, are important methods to understand the mechanism of membrane-associated transporter protein (MATP) at the atomic level, which helps in the field of pharmacogenomics,^28^ and highlight the dynamic behavior of the key regions involved in protein–protein interactions such as those related to proliferating cell nuclear antigen (PCNA).^29^ This protein nearly exists in every form of life and participates in the mismatch repair process as the principal conductor at the replication fork in which the computational screening contributes to providing the essential structural and functional features required for the DNA-mismatch repair process.^30^ Earlier, this approach allowed the selection of Herceptin, an antibreast cancer drug, as an effective drug for the molecular target of the HER2 receptor, even with the most deleterious nsSNP.^31^ In another study on malaria, molecular docking analysis identified the best selective inhibitor against the crystal structure of *Plasmodium falciparum* LDH with no effect on human LDH.^32^

The traditional consumption of wild edible species with many pharmacological effects is rapidly gaining popularity and highlights the value of these natural products.^33^ To establish solid scientific facts for folk medicine practices and the inclusion of species like lichen in daily diet, more studies are necessary as the scientific evidence in favor of these practices is sometimes limited.^34^ Therefore, the purpose of this study is to investigate the potential anticancer effects of *E. prunastri* extracts, known as therapeutic spices in Algeria. In particular, this study focused on the cytotoxic activities of *E. prunastri* on human colon cancer (HT-29), human prostate cancer (PC-3), human non-small cell lung cancer cells (A549), and healthy human cells (CCD18-Co). Furthermore, the antioxidant and anti-inflammatory activities of *E. prunastri* were also evaluated, as these properties may contribute to its potential as an anticancer agent in addition to quantifying its phenolic compounds. To the best of our knowledge, no such report has been found to date, despite recent studies. This study provides the increasing mass of scientific evidence on the possible curative benefits of folk medicine practices and highlights the potential therapeutic effects of spice consumption, used by local people in Algeria as a remedy ingredient such as *E. prunastri*.^20^

## 2 Materials and Methods

### 2.1 Plant Material

**Figure 1:**
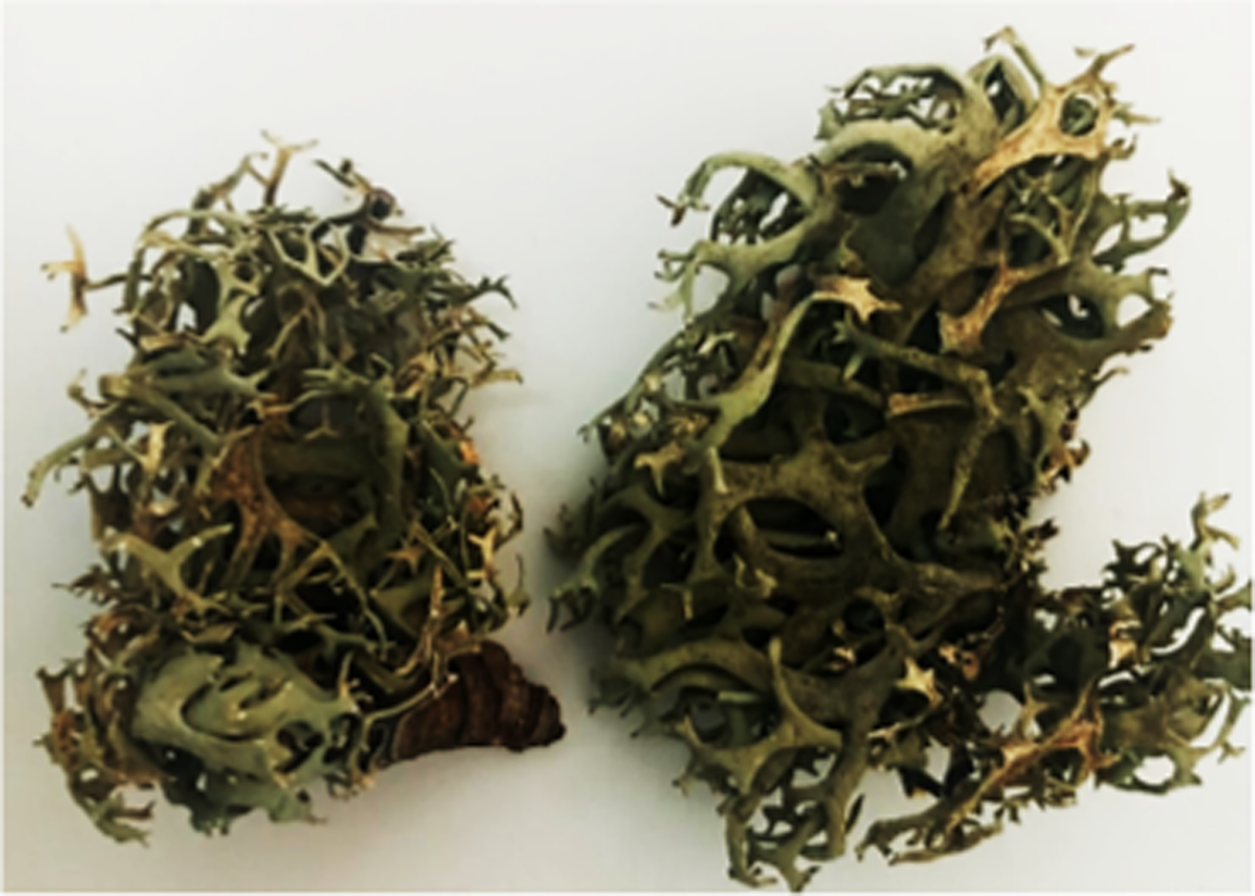
Thallus of *E. prunastri* (L.) Ach. (Galaxy Note20 Ultra 5G Camera).

Lichen samples of *E. prunastri* (L.) Ach. were collected from the tree bark growing in windy, lit, and humid areas of Aïn Defla, Algeria, in December 2021. Botanical identification was conducted, and a voucher specimen (Voucher No. LRSBG/AB/20/158) was deposited in the Department of Biology (University of Mascara, Algeria). The fresh thallus was prewashed and dried for several days at ambient temperature (20–30 °C) in the laboratory (Figure 1). After 10 days, the sample was crushed using a grinder (type IKA M20 Laboratory universal grinder), and the recovered powder was stored in a black container.

### 2.2 Chemicals

MTT solution (M6494: Applichem, USA), methanol (106009), ethanol (E7023), hydrochloric acid (30721), acetic acid (A6283), ferric chloride (F2877), sulfuric acid (07208), magnesium ribbon (13103), Hager’s reagent (0792A 00125), sodium hydroxide (1310732), 2,2-diphenyl-1-picrylhydrazyl (DPPH; D9132), phosphomolybdate reagent (417895000), phosphate buffer (P5244), and a 30% H_2_O_2_ solution (H1009) were obtained from commercial sources (Sigma-Aldrich, Germany).

### 2.3 Preparation of Plant Extracts

Aqueous and methanolic extracts were used to obtain the *E. prunastri* extracts. Briefly, for the aqueous extract, 20 g of thallus powder and 200 mL of distilled water were combined and heated to 100 °C with continuous stirring for 20 min. The mixture was cooled for 10 min and filtered, and then the remaining material was re-extracted twice following the same process. All the extracts obtained were combined, concentrated, lyophilized, and stored at 4 °C until further analysis.^35^

The maceration process of the methanol extract was carried out in accordance with Sioud et al. 40 g of dried thallus was macerated with 400 mL of methanol for 7 days. The mixture was continuously stirred daily at room temperature and stored overnight at 4 °C. The extracts were concentrated using a rotary evaporator at low pressure at 40 °C, and the obtained crude extracts were stored at 4 °C until further investigation.^36^

### 2.4 Screening for Total Phenolic, Flavonoid, and Tannin Contents

Phytochemical screening of *E. prunastri* extracts was performed using standard methods adapted from previous studies based on color and precipitation reactions to reveal the presence of alkaloids, saponins, steroids, flavonoids, reducing sugars, terpenoids, and tannins.^37−40^ On the other hand, total tannin (TTC), flavonoid (TFC), and phenolic (TPC) contents were determined using colorimetric methods, according to previous studies. The results were reported as standard equivalent based on the amount of dried extract (mg standard/g dry extract).^38,41−43^

### 2.5 HPLC-DAD Analysis

The chemical profile was analyzed by HPLC-DAD, according to procedures by Çayan et al. and Küçükaydın et al. under standard conditions.^44,45^ The extracts were dissolved in an 80:20 mixture of water and methanol. Following filtration using a single-use LC filter disk (0.20 μm), the sample was separated using an Inertsil ODS-3 reversed-phase C_18_ column (Nanologica) (5 μm, 250 mm × 4.6 mm i.d). A 20 μL of the sample was injected at a flow rate of 1.0 mL/min. A PDA detector was used to monitor the phytochemicals at 280 nm. The phytochemicals were then identified based on their retention times and UV data by comparing them with reference compounds. To identify and quantify the phenolic compounds, the samples were handled, and a calibration curve was generated by injecting known concentrations of several reference chemicals (3-hydroxy benzoic acid, 6,7-dihydroxy coumarin, apigenin, caffeic acid, catechin, chlorogenic acid, p-coumaric acid, chrysin, coumarin, ellagic acid, ferulic acid, gallic acid, hesperetin, luteolin, myricetin, protocatechuic acid, pyrocatechol, p-hydroxy benzoic acid, syringic acid, taxifolin, rutin, rosmarinic acid, vanillin, quercetin, trans-cinnamic acid, and kaempferol). The results were given as mg/g of dry weight (dw).^46^

### 2.6 Antioxidant Activity

The antioxidant activities (DPPH and H_2_O_2_ radical scavenging, reducing power (FRAP), and total antioxidant capacity) were determined using previous methods, and the results were expressed as the equivalents of the standards.^47^

#### 2.6.1 Total Antioxidant Capacity (TAC) Assay

The total antioxidant capacity (TAC) of both extracts, as well as ascorbic acid (as positive control) (25, 50, 100, 250, and 500 μg/mL), were measured using the phosphomolybdate assay. In brief, 3 mL of phosphomolybdate reagent was added to 300 μL of the samples and incubated in a water bath at 95 °C for 90 min. After cooling the mixture to room temperature, absorbance was recorded at 765 nm. The results were expressed as mg/g of standard equivalent (mg/g AAE).^48^

#### 2.6.2 DPPH Radical Scavenging Activity

The DPPH free radical scavenging activity was assessed using the method described by Uysal et al. with slight modifications.^48^ In summary, DPPH solution (0.1 mM) was mixed with the extract (1 mL) and incubated for 16 min. The absorbance of each sample was then measured at 517 nm. Ascorbic acid was used as the positive control.

#### 2.6.3 Reducing Power (RP) Assay

Reducing power was measured by using a potassium ferricyanide assay. The reaction mixture consisted of 2.5 mL of the extract, phosphate buffer (2.5 mL, 0.2 M, pH 6.6), and potassium ferricyanide (1.0 mL, 10 mg/mL). The mixture was incubated at 50 °C for 20 min, and then 1.0 mL of trichloroacetic acid (100 mg/mL) was added. The solution was then centrifuged for 10 min at 650 rpm. The absorbance was measured at 700 nm after the supernatant (5 mL) was combined with 1 mL ferric chloride (1.0 mg/mL) and 5 mL distilled water. The same steps were performed for ascorbic acid (positive control), and the results were expressed in mg AAE/g extract.^49^

#### 2.6.4 Hydrogen Peroxide (H_2_O_2_) Assay

By using the method of Ruch et al.,^50^ the capacity of extracts to scavenge hydrogen peroxide was determined at different concentrations (25–500 mg/mL) of samples (methanolic and aqueous) and the positive control (ascorbic acid). 3 mL aliquot of previously prepared work solution of hydrogen peroxide (4 mM) was mixed with 100 μL of extracts/standard. After a reaction time of 10 min, the absorbance of hydrogen peroxide was measured at 230 nm against a blank solution.

### 2.7 Anti-inflammatory Activities

#### 2.7.1 HRBC Membrane Stabilizing Assay

The HRBC suspension was prepared by mixing equal volumes of blood (O+) and ELSIVER solution, followed by centrifuging at 3000 rpm for 10 min. The packed cells were washed three times with an equivalent volume of isosaline (PBS, pH 6.3). The process was ended by reconstructing the pellet as a 10% v/v suspension in PBS.^51^ The results were compared with those obtained using diclofenac sodium (s-DCF) as a positive control.

##### 2.7.1.1 Hypotonic Solution-Induced Hemolysis

The hypotonic solution-induced hemolysis assay was performed according to Azeem et al.^52^ The reaction mixture consisted of 1 ml of varying extract concentrations (25, 50, 100, 250, and 500 μg/mL), 0.5 mL of HRBC suspension, 1 mL of phosphate buffer, and 2 mL of hyposaline (0.25% w/v NaCl). After 30 min of incubation at 37 °C, the mixture was centrifuged for 20 min at 3000 rpm. Using spectrophotometry, the hemoglobin concentration of the supernatant solution was determined at 560 nm. Using this technique, the ability of *E. prunastri* to stabilize the red blood cell membranes was assessed in comparison with s-DCF.

##### 2.7.1.2 Heat-Induced Hemolysis

This assay was performed as described previously.^53,54^ 1 mL of 10% RBC suspension was added to 1 mL of the sample at different concentrations (25–500 μg/mL). All of the centrifuge tubes containing the reaction mixture were placed in a water bath at 56 °C for 30 min. After cooling, the mixture was centrifuged for 5 min at 3000 rpm. The absorbance of the supernatant was measured at 560 nm.

#### 2.7.2 Protein Denaturation Inhibition Assay

The potential of the extracts to inhibit protein denaturation was evaluated using bovine serum albumin, egg albumin, and casein as substrates.^55^

##### 2.7.2.1 Bovine Serum Albumin (BSA) Denaturation Assay

In the bovine serum albumin (BSA) denaturation assay, 0.05 mL of the extract at different doses (50, 100, 250, and 500 μg/mL) was combined with 0.45 mL BSA (0.5% w/v aqueous solution). The resulting mixtures were incubated for 20 min at 37 °C, and then for 3 min at 57 °C, and the temperature was adjusted. After cooling, 2.5 mL of pH 6.6 phosphate buffer was added and the absorbance at 255 nm was determined. The negative control consisted of 0.45 mL of 0.5% BSA and 50 μL distilled water.^56,57^

##### 2.7.2.2 Egg Albumin Denaturation Inhibition Assay

A previously validated method by Sunmathi et al. was used to investigate the denaturation inhibition capacity of both extracts and s-DCF at varying concentrations (50, 100, 250, 500 μg/mL).^51^ The reaction mixture contained 0.2 mL of egg albumin (0.5% w/v aqueous solution), 2.8 mL of PBS (pH = 6.4), and 2 mL of the extract. After 15 min of incubation at 37 °C, the reaction mixture was heated to 70 °C for 5 min. After cooling, the absorbance was measured at 660 nm.

##### 2.7.2.3 Proteinase Inhibition Assay

The inhibitory activity of trypsin enzymatic activity was tested, as previously described.^46^ The reaction mixture (2 mL) contained 1 mL of 20 mM Tris HCl buffer (pH 7.4), 1 mL of the test sample at various concentrations, and 0.06 mg of trypsin. The mixture was incubated for 5 min at 37 °C. After adding 1 mL of 0.8% (w/v) casein, the mixture was incubated for 20 min. To end the reaction, 2 mL of 70% perchloric acid was added. After centrifugation, the absorbance of the supernatant was read at 210 nm against the buffer as a negative control, while s-DCF was utilized as the positive control.

### 2.8 Anticancer activity

#### 2.8.1 Cell Culture

Colon, prostate, and lung cancer cell lines (HT-29, PC-3, A549) and healthy human colon fibroblast (CCD18-Co) cell lines were cultured in RPMI (Roswell Park Memorial Institute) growth medium containing 10% fetal bovine serum. The cells were cultured at 37 °C in a CO_2_ incubator with 5% CO_2_ and 95% relative humidity.

#### 2.8.2 MTT Assay

To measure the cytotoxic ability of *E. prunastri* extracts against three colon, prostate, and lung cancer cell lines (HT-29, PC-3, A549) and human nontumor (CCD18-Co) cell lines, the MTT colorimetric assay was used.^58^ In brief, cells were plated in 96-well plates in triplicate at a density of 10,000 cells/well. Each cell line was cultured at 37 °C in a humidified environment with 5% CO_2_ and 95% air. After the extracts were added to the wells at four different final concentrations ranging from 200 to 25 μg/mL, the wells were incubated for 24, 48, and 72 h. Subsequently, 10 μL of MTT reagent (Applichem) in phosphate-buffered saline (PBS) was added. The medium was carefully disposed of after the 4 h incubation period, and DMSO (100 μL) was added to dissolve the formazan that developed in the cells. Using a microplate reader, absorbance was measured at 540 nm following a 10 min incubation period.^58^ The negative control group cells were left untreated. A well containing doxorubicin against PC-3, cisplatin against A549, and HT-29 cancer cell lines served as positive controls. The IC_50_ value was determined according to the time curve (24, 48, and 72 h), which gave the highest % cytotoxicity values. The IC_50_ values were determined using an AAT Bioquest IC_50_ calculator,^59^ and the results were compared with the IC_50_ values calculated using a series of dose–response data, in which extract concentrations and cytotoxic activity (% inhibition) were plotted.

### 2.9 Statistical Analysis

Data are presented as the mean ± standard deviation (*n* = 3). Excel PRO 2019 software was used to calculate the antioxidant, anti-inflammatory, and cell survival rates. GraphPad Prism version 8 was used for statistical analysis including one-way ANOVA followed by Tukey’s multiple comparison tests. *p* < 0.05 was considered statistically significant. The values of IC_50_ were determined, as previously described from the regression curves obtained from the percentages of inhibition or absorbance at different concentrations.

## 3 Results and Discussion

### 3.1 Extraction Yield and Quantification of Phenolic Compounds

As shown in Table 1, both aqueous and methanolic extracts of *E. prunastri* showed almost similar extraction yields of 9.65 ± 0.007 and 11.08 ± 0.006%, respectively. The relatively higher extraction yield shown by the methanolic extract corroborates the findings of Bézivin et al., who stated that most lichen compounds are found in the methanol extract.^60^

**Table 1:** Extraction Yield, Total Phenolic, Flavonoid, and Tannin Contents of *E. prunasti* Extracts^a^

*E. prunastri*	yield %	phenols, μg GAE/mg	flavonoids, μg QEE/mg	tannins, μg CE/mg
aqueous	10.65 ± 0.007	199.17 ± 0.009****	127.84 ± 0.000	106.50 ± 0.003****
methanolic	11.08 ± 0.006	280.83 ± 0.002****	148.07 ± 0.000	201.50 ± 0.000****

a Results were considered highly significant at ****p < 0.0001.

**Figure 2:**
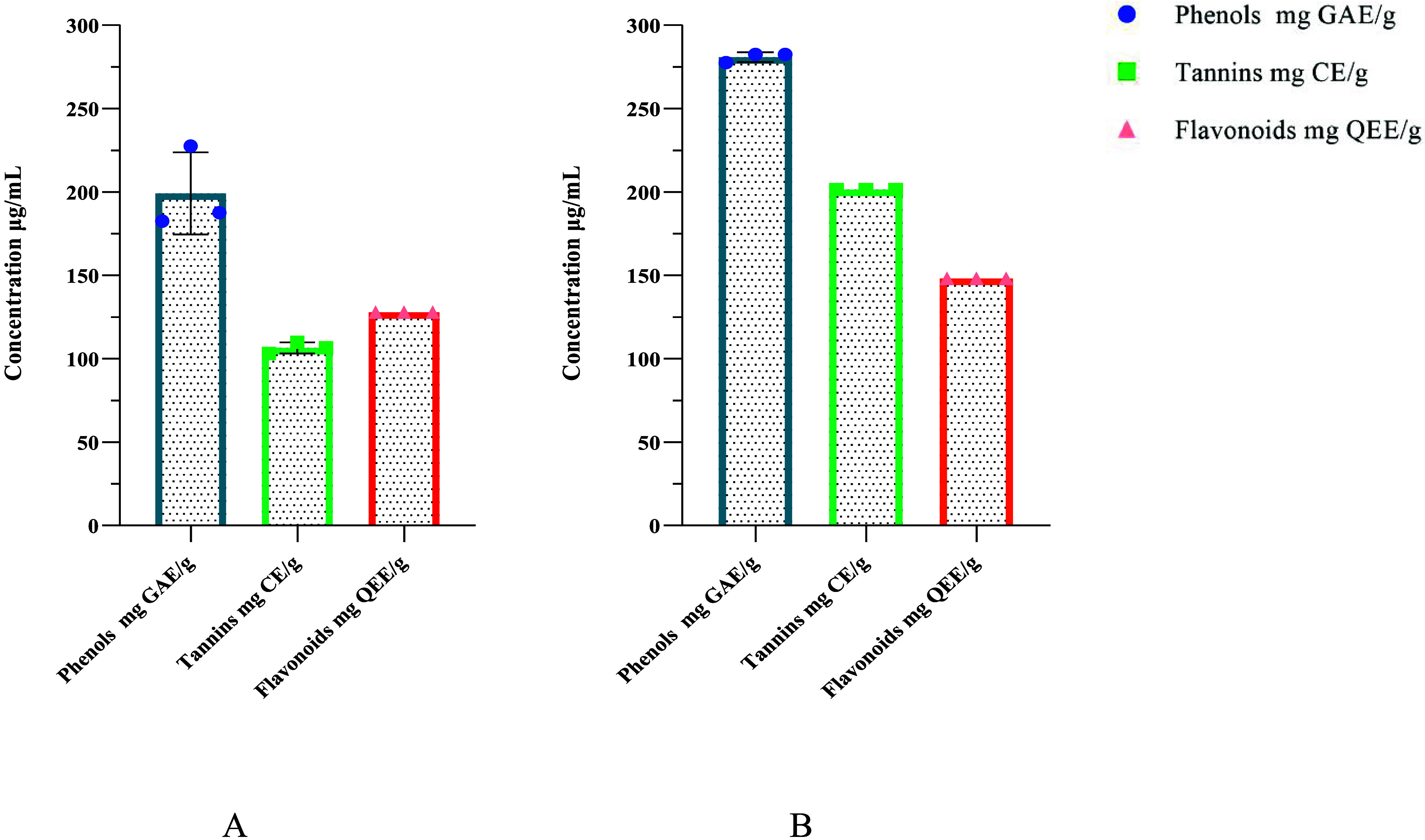
Phytochemical quantitative comparison of *E. prunastri* extracts: (A) aqueous extract and (B) methanol extract.

Moreover, our results (Figure 2) showed that the methanolic extract contained higher phenolic, flavonoid, and tannin content than the aqueous extract (Figure 2A). The TPC and TTC of the methanolic extract were determined to be 280.83 ± 0.002 μg GAE/mg and 201.50 ± 0.000 μg CE/mg, whereas those of the aqueous extract were 199.17 ± 0.009 μg GAE/mg and 106.50 ± 0.003 μg CE/mg, respectively, with significant differences at *p* < 0.0001. Similarly, the TFC of the methanolic and aqueous extracts were 148.07 ± 0.000 and 127.84 ± 0.000 μg QEE/mg, respectively. The TPC and TFC of the methanol extract of *E. prunastri* were 373 ± 4.2 mg (CE/100 g Dw) and 3585 ± 69.30 (mg GAE/100 g Dw).^22^ Although TPC, TFC, and TTC contents in the methanolic extract of *E. prunastri* were found to be lower than those in the acetone extract,^11^ the methanolic extracts of lichen species showed higher TPC, TFC, and TTC contents than the aqueous extracts.^60^ Aoussar et al. reported the yields (% w/w) of dried extracts of *E. prunastri to be* 3.7% (dichloromethane), 10.1% (acetone), and 8% (methanol), respectively.^11^ The phenolic content of the acetone extract of *E. prunastri* was 34.05 ± 1.065 μg PE/mg.^61^ The methanol extract of *E. prunastri* was found to have a total phenolic content of 80.73 mg GA/g and a total flavonoid content of 27.46 mg Ru/g.^62^ Differences may be explained by a reduced hydrosolubility of components produced by lichens, as suggested by previous report.^63^

### 3.2 Characterization of Phenolic Compounds

Preliminary phytochemical screening showed the presence of different phytochemicals. As can be seen from Table 2, the lichen aqueous and methanolic extracts contained phenols, saponins, flavonoids, tannins, glycosides, quinones, terpenoids, coumarins, and alkaloids. Moreover, anthraquinones, resins, steroids, phytosteroids, and phlobatannins were not detected in both of the extracts. On the other hand, the methanolic extracts contained gallic tannins.

**Table 2:** Phytochemical Screening of *E. prunastri* Extracts^a^

**samples**	**Ep.aq**	**Ep.met**
anthocyanins	++	++
iridoids	++	++
quinones	++	+
coumarins	++	++
anthraquinones	–	–
phlobatannins	–	–
glycosides	+	+
saponins	+	+
steroids	–	–
phytosteroids	–	–
flavonoids	++	+++
terpenoids	++	++
phenols	++	++
protein/AA	+	–
reducing sugars	+	–
alkaloids	+	+
saponosids	+	+
cardiac glycosides	+	+
resins	–	–
tannins	+	+
gallic tannins	–	+
catechin tannins	–	–

a +++ Very abundant; ++ abundant; + slightly abundant; – not detected.

Phenolic components are potential antioxidants owing to their ability to scavenge free radicals.^64−66^ By comparison of 26 standards, HPLC-DAD analysis allowed us to identify 14 phenolic compounds. Among them, rosmarinic acid (79.17; 14.35 mg/g), caffeic acid (46.52; 5.11 mg/g), *trans*-cinnamic acid (29.23; 14.42 mg/g), chlorogenic acid (23.68; 5.27 mg/g), quercetin (11.34; 3.87 mg/g), *p*-coumaric acid (8.16; 3.67 mg/g), rutin (6.79; 2.28 mg/g), and apigenin (6.93; 1.37 mg/g) were the main chemicals in the methanolic and aqueous extracts, respectively. Additionally, luteolin, kaempferol, and myricetin were exclusively detected in the methanolic extract in small amounts but not in the aqueous extract. The latter was characterized by the presence of pyrocatechol. Moreover, it appears that the major phenolic constituents belong to phenylpropanoid and flavonoid classes (Table 3).

**Table 3:** Phenolic Composition of *E. prunastri* Extracts by HPLC-DAD (mg/g extract)

**no**	**phenolic compounds**	**RT (min)**	**EP-Met**	**EP-Aqu**	**main class**
**1.**	pyrocatechol	11.04		0.91	benzenediols
**2.**	rutin	25.30	6.79	2.28	flavonoids
**3.**	myricetin	27.35	1.98		
**4.**	quercetin	30.43	11.34	3.87	
**5.**	luteolin	31.70	1.98		
**6.**	kaempferol	33.21	2.56		
**7.**	apigenin	33.77	6.93	1.37	
**8.**	gallic acid	5.70	3.94	2.05	phenolic acids
**9.**	3-hydroxy benzoic acid	15.98	3.61	0.98	
**10.**	chlorogenic acid	12.35	23.68	5.27	phenylpropanoids
**11.**	caffeic acid	15.09	46.52	5.11	
**12.**	*p-*coumaric acid	20.56	8.16	3.67	
**13.**	rosmarinic acid	26.77	79.17	14.35	
**14.**	*trans*-cinnamic acid	31.33	29.23	14.42	

This is the first report of phenolic compounds in *E. prunastri,* revealing the presence of these secondary metabolites, with rosmarinic acid as the predominant one in this lichen species. Consequently, there are no previous studies supporting our findings. Arup et al. detected the presence of usnic acid, evernic acid, and atranorin in the acetone extract of *E. prunastri* using the HPTLC method.^67^ Regardless of these, Kosanić et al. detected the presence of usnic acid, evernic acid, physodic acid, chloroatranorin, and atranorin in the acetone extract of *E. prunastri* using the HPLC–UV method.^61^ Likewise, it has been reported that methyl lecanorate, evernic acid, usnic acid, atranorin, and chloratranorin were found in abundance in the dichloromethane and acetone extracts of *E. prunastri*, while they were less detected in the methanolic extract.^11^ Moreover, HPLC-DAD analysis showed that acetone was found to be more effective than methanol for extracting atranorin, evernic acid, and usnic acid from *E. prunastri*.^68^ Kahriman et al. reported that the essential oil of *E. prunastri* was rich in β-pinene, α-pinene, limonene, α*-*phellandrene, camphene, and p-cymene.^69^

### 3.3 Antioxidant Activity

**Figure 3:**
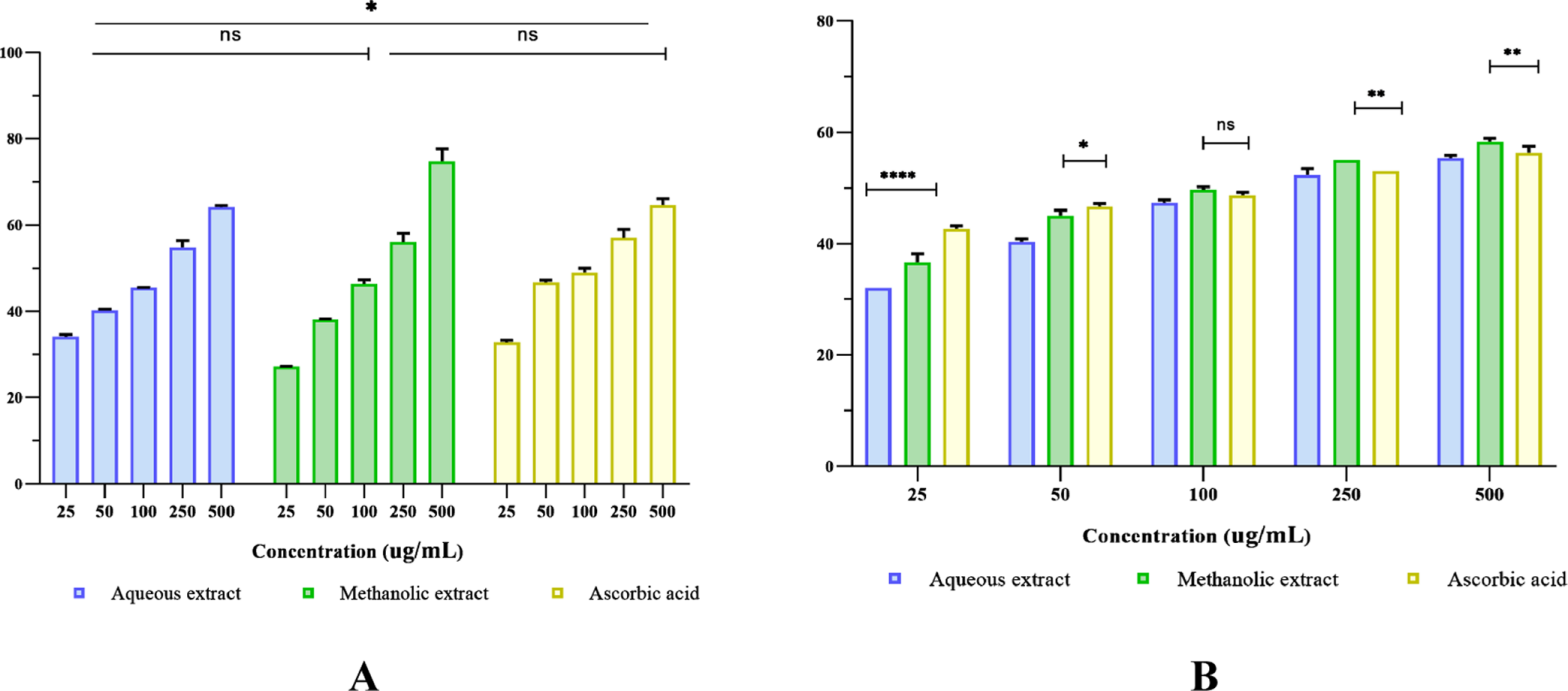
Antioxidant activities of aqueous and methanolic extracts of E. prunastri compared to ascorbic acid. (A) DPPH scavenging activity, (B) H_2_O_2_ scavenging capacity. ****: *p* < 0.0001, **:*p* < 0.01, *:*p* < 0.05, and ns: *p* > 0.05.

As shown in Table 4, the *E. prunastri* methanolic extract exhibited a higher TAC compared to ascorbic acid (positive control). In fact, the methanolic extract had a TAC value of 13.82 ± 0.04 mg AAE/g followed by that of ascorbic acid (11.05 ± 0.01 mg AAE/g; *p* < 0.01), whereas that of the aqueous extract was significantly lower (6.75 ± 0.03 mg AAE/g (*p* < 0.001)). Similarly, our results (Figure 3A) revealed that the *E. prunastri* methanolic extract exerted an important DPPH scavenging activity that was higher than that of the aqueous extract and ascorbic acid. At 500 μg/mL, a higher DPPH scavenging activity (76.04 ± 0.01%) was exhibited by the methanol extract, while the positive control and aqueous extract were found to be 64.67 ± 0.03 and 63.91 ± 0.01%, respectively. Accordingly, the methanolic extract had the lowest IC_50_ value of 200 ± 0.048 μg/mL, which was similar to that of ascorbic acid (200 ± 0.004 μg/mL, not significant at *p* > 0.05). The free radical scavenging potential is regarded as an important antioxidative mechanism of natural products.^68^ Our findings revealed that the methanolic extract exhibited a higher DPPH scavenging activity and was comparable to that of the positive control. These results are contrary to those of previous studies. Indeed, the acetone extract of *E. prunastri* showed a weak DPPH scavenging activity (IC_50_ = 663.12 lg/mL) compared to ascorbic acid (IC_50_ = 6.42 lg/mL).^61^ Likewise, the effect of the *E. prunastri* methanol extract was clearly lower than those of the ascorbic acid and Trolox.^11,70^

**Table 4:** Antioxidant Activities of *E. prunastri* Extracts Compared With Ascorbic Acid (Expressed by IC_50_ (μg/mL) and mg AAE/g ± SD)^a^

	**IC**_**50**_**(μg/mL) ± SD**		**mg** AAE/g	
**samples**	DPPH	H_2_O_2_	TAC	FRAP
aqueous extract	217 ± 0.035*	300 ± 0.577****	6.75 ± 0.030***	3.47 ± 0.001****
methanolic extract	200 ± 0.048^ns^	200 ± 0.000****	13.82 ± 0.040**	4.17 ± 0.001*
ascorbic acid	200 ± 0.004	167 ± 0.000	11.05 ± 0.011	4.15 ± 0.000

a ****: *p* < 0.0001, ***:*p* < 0.001, **:*p* < 0.01, *:*p* < 0.05, and ns: *p* > 0.05.

Consistent with its stronger ability to scavenge DPPH radicals, the methanolic extract possessed a significant reducing power of 4.17 ± 0.001 mg AAE/g, which exceeded that of the aqueous extract (3.47 ± 0.001 mg AAE/g; *p* < 0.0001), whereas that of ascorbic acid was 4.15 ± 0.000 mg AAE/g (Table 4). The presence of polyphenols in the extracts, which can transform free radicals into more stable and unreactive species, may be responsible for their scavenging ability.^70^ Nevertheless, previous studies have shown that the reducing power of different extracts of *E. prunastri* was observed to be less effective than the other species and ascorbic acid.^11,70^

The H_2_O_2_ scavenging capacity of the *E. prunastri* methanolic extract was higher than that of ascorbic acid (*p* = 0.0083) at high doses (Figure 3B). The methanolic extract showed the best antioxidant effect (58.33 ± 0.001%; IC_50_ = 200 ± 0.000 μg/mL), followed by ascorbic acid (56.33 ± 0.005%; IC_50_ = 167 ± 0.000 μg/mL), whereas the scavenging effect of the aqueous extract was determined to be the lowest with an IC_50_ of 300 ± 0.577 μg/mL (Table 4).

Here, we demonstrate that *E. prunastri* has strong antioxidant activity, as assessed *in vitro* against various oxidative systems, with significantly enhanced antioxidant potential shown by the methanolic extract. This discrepancy could result from the difference in the capacities of the two solvents to extract the bioactive substances.^71,72^ In fact, it has been demonstrated that phenolic compounds of lichens seem to be responsible for their antioxidant potential. Earlier, Aldoghachi et al. reported the antioxidant potential of rosmarinic acid.^74^ Furthermore, caffeic acid as an *E. prunastri* metabolite showed an important antioxidant effect and other biological activities. Considering the benefits of high stability and extraction from natural sources, this phenolic acid has been found to be a potent antioxidant agent that could rival both ascorbic acid and trolox.^73,75^ Recently, several studies have emphasized the antioxidant effects of phenolic acids such as chlorogenic acid, kaempferol, and apigenin acting as free radical scavenging agents.^76−78^ Our study herein confirms that the methanolic extract had the highest phenolic compounds and the highest antioxidant capacities. The results corroborate those of the previous reports.^79−82^ Despite numerous studies exploring the antioxidant potential and other pharmacological activities of lichen phenolic compounds, the latter have not been sufficiently determined.^83^

### 3.4 Anti-inflammatory Activity

Assays for protein denaturation inhibition, antiprotease activity, and HRBC membrane stabilization were used to assess the anti-inflammatory properties of the *E. prunastri* extracts. To evaluate and compare the anti-inflammatory activity, s-DCF was used as a positive control.

#### 3.4.1 HRBC Membrane Stabilization Assay: Hypotonicity and Heat-Induced Hemolysis

**Figure 4:**
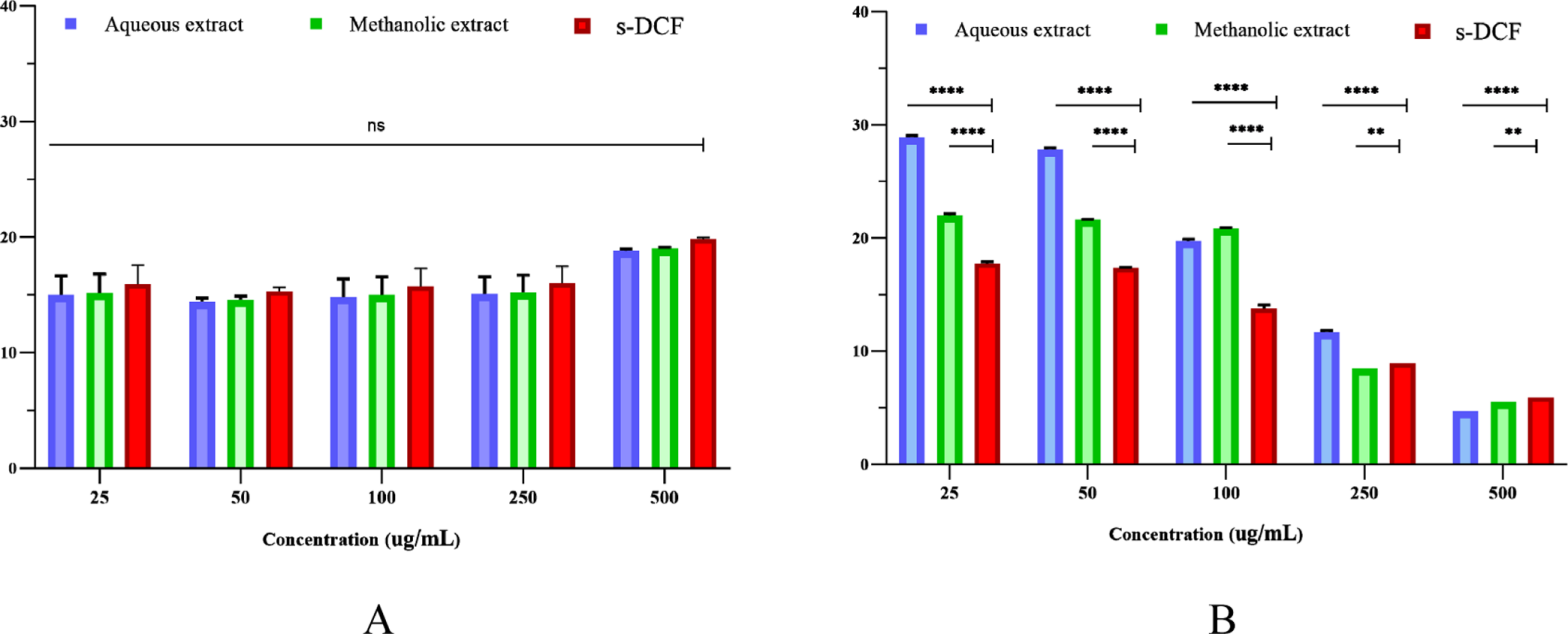
HRBC membrane stabilization activity (A) and hemolysis percentage (B) of *E. prunastri* extracts compared with s-DCF. ns: *p* > 0.05, **:*p* < 0.01, and ****:*p* < 0.0001.

As displayed in Figure 4A, no significant difference was observed neither between the methanolic and aqueous extracts nor between the extracts and s-DCF (*p* > 0.05). The activity was significant at the highest concentration (500 μg/mL) for all samples, resulting in HRBC stabilization rates of 19.73 ± 0.012, 18.98 ± 0.030, and 18.82 ± 0.011% for s-DCF, methanolic, and aqueous extracts, respectively.

Furthermore, our results showed that both *E. prunastri* extracts and s-DCF exhibited dose-dependent antihemolytic effects. Although s-DCF (25–250 μg/mL) had the best antihemolytic activity, both extracts had the highest protective effect at 500 μg/mL (Figure 4B). In fact, the aqueous and methanolic extracts reduced hemolysis to 4.67 ± 0.00 and 5.50 ± 0.00 μg/mL, respectively.

Our findings concur with those of the previous studies that demonstrated the anti-inflammatory activity of lichens. In fact, *E. prunastri* showed considerable effectiveness as an anti-inflammatory drug, either by maintaining the lysosomal membrane or by preventing the release of lysosomal enzymes.^84^ The existence of phytochemicals, such as flavonoids and phenolics, might be responsible for this. A previous study found that the extract of other lichen species decreased the p-IκBα and p-65 levels, resulting in the inhibition of NF-κB signaling and a decrease in the expression of cytokines and mediators such as TNF-α, IL-6, and NO. The study concluded that the constituents of lichen extracts are capable of blocking inflammation via NF-κB signaling.^85^ On the other hand, an excessive fluid buildup inside the cells might cause the RBC membrane to rupture, which can result in catastrophic damage from lipid peroxidation caused by free radicals. Inflammatory mediators cause fluids and serum proteins to seep into tissues. This process could be prevented by stabilizing the blood membrane. The extracts from *E. prunastri* may maintain RBC membranes by inhibiting the release of lytic enzymes and other potent inflammatory mediators.^86^

#### 3.4.2 Antidenaturation and Proteinase Inhibition Activity

**Figure 5:**
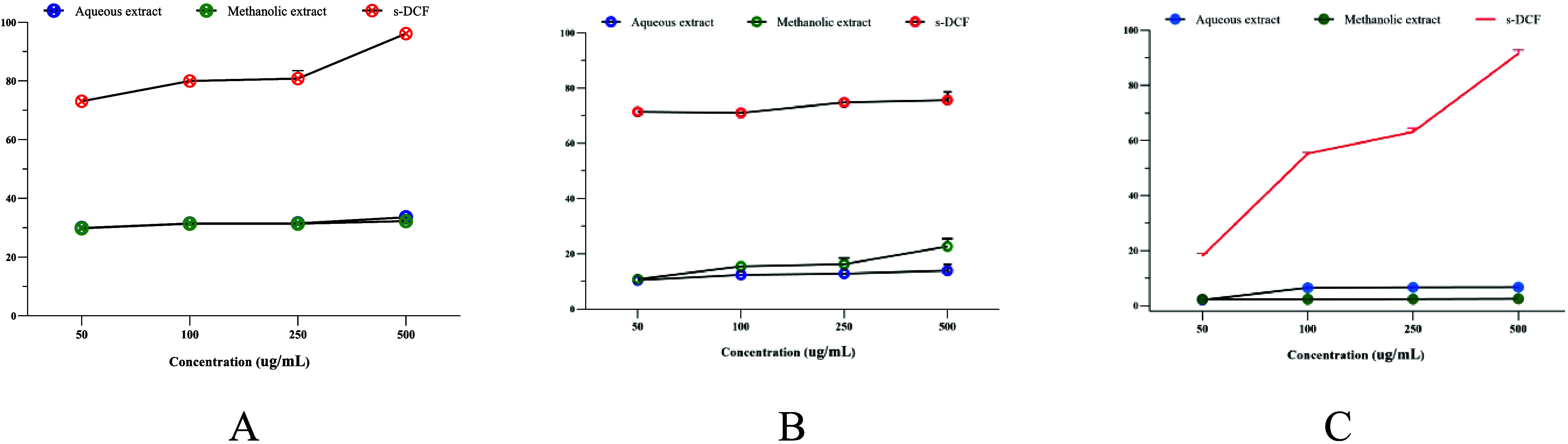
Inhibitory activities of denaturation of bovine albumin (A), egg albumin (B), and proteinase inhibition (C) of *E. prunastri* extracts compared to s-DCF.

We then investigated the in vitro anti-inflammatory effects of *E. prunastri* against the denaturation of bovine and egg albumin. The results are shown in Figure 5. Both extracts exhibited antidenaturation activities. The highest preventive effect against denaturation of bovine albumin was reached at 500 μg/mL, and was found to be 33.55 ± 0.02 and 32.25 ± 0.03% for the aqueous and methanolic extracts, respectively (Figure 5A). However, s-DCF significantly suppressed denaturation with the greatest value of 96.09 ± 0.00% (*p* < 0.0001). At the same concentration (500 μg/mL), the aqueous and methanolic extracts of E. prunastri showed maximum antidenaturation values of 13.86 ± 0.28 and 22.67 ± 1.15%, respectively for egg albumin (Figure 5B). However, the preventive effect of s-DCF was found to be significantly higher (75.60 ± 0.31%) than that of both extracts (*p* < 0.0001). This was further confirmed by comparing their IC_50_ values (Table 5). Furthermore, we noticed that *E. prunastri* showed a modest proteinase inhibitory effect ranging from 2.50 ± 0.08 to 6.74 ± 0.04%, whereas that exerted by s-DCF was found to be significantly increased (*p* < 0.0001) with the highest value of 51 ± 0.10% at 500 μg/mL (Figure 5C).

**Table 5:** IC_50_ Values of Anti-Inflammatory Activity of *E. prunastri* Extracts Compared to s-DCF * (*p* < 0.05)

	**IC**_**50**_**(μg/mL) ± SD**		
**samples**	HRBC	protein	proteinase
aqueous extract	>500	>500	>500
methanolic extract	>500	>500	>500
s-DCF	>500	440 ± 0.13*	350 ± 0.10*

Thus, the anti-inflammatory effect of *E. prunastri* may be attributed to its ability to reduce HRBC hemolytic damage. However, no effect was recorded on proteinase inhibition, although both extracts showed a modest effect on protein denaturation. It is worth noting that the lack of research on the anti-inflammatory activity of *E. prunastri* makes it difficult to compare the present findings with those of previous studies. Indeed, earlier investigations have focused on the mosde of action of anti-inflammatory properties of phenolic compounds revealed therein. These molecules can reduce the inflammation process with minimal or null side effects.^87^ In fact, phenolic compounds such as myricetin, quercetin, kaempferol, luteolin, and apigenin were previously reported to exhibit potential inhibition of NO production and iNOS protein and mRNA expression.^88,89^ Moreover, all of the active compounds found in edible tropical species inhibited the activation of nuclear factor-κB (NF-κB), which is a significant transcription factor for iNOS.^90^ In addition, an in vivo study investigated the effect of rosmarinic acid on local inflammation (carrageenin-induced paw edema model in the rats) and evaluated the protective effect in rat models of systemic inflammation: liver ischemia-reperfusion (I/R) and thermal injury models. Rosmarinic acid, the main compound in *E. prunastri*, reduced paw edema, transaminases (AST and ALT), and LDH in addition to modulation of NF-κB and metalloproteinase-9.^91^ Protein denaturation mechanism includes electrostatic hydrogen modification and hydrophobic and disulfide bonding, leading to the production of autoantigens in some inflammatory conditions.^92^ Thus, by inhibition of protein denaturation, the inflammatory process can be blocked. Some drugs, NSAIDs, which also inhibit the COX enzyme, reduce protein denaturation.^93^ However, they can lead to various side effects of ulceration, bleeding, perforation, and obstruction.^94^ Regarding the modest anti-albumin denaturation activity, *E. prunastri* may attenuate inflammation by suppressing expressions of cyclooxygenase enzyme. Since lichens contain a high concentration of biologically active compounds that have demonstrated potential as antioxidants and anti-inflammatory agents and have been more widely accepted in traditional herbal treatments.^95,96^ Besides, we previously reported the use of *E. prunastri* as a treatment for infectious ailments.^20^ Consequently, its phenolic compounds could be the main contributors to the anti-inflammatory effect.

### 3.5 Anticancer Effect

Recently, several reports have evaluated the anticancer ability of lichen extracts along with the identification of active metabolites.^97^ To determine the cytotoxic potential of *E. prunastri* extracts, we treated three different carcinoma cells with the aqueous and methanolic extracts using the MTT method. Cell lines (HT-29, PC-3, A549, and CCD18-Co) were provided by Dr. Aydın Demiray (Pamukkale University, Faculty of Medicine, Denizli-Turkey). Accordingly, the methanolic extract had the lowest IC_50_ values of 100 ± 0.04, 146 ± 0.05, and 112 ± 0.06 μg/mL (*p* < 0.05) against HT-29, PC-3, and A549 cell lines, respectively, after 72 h. The aqueous extract had an IC_50_ > 200 μg/mL for all of the tested cell lines. Interestingly, both methanolic and aqueous extracts of *E. prunastri* had an IC_50_ > 200 μg/mL against the CCD18-Co nontumor cell line (Table 6). The cytotoxic effect of cisplatin on HT-29 and A 549, which were positive controls, were determined as IC_50_ values of 4.97 ± 0.01 and 2.95 ± 0.01 μg/mL, respectively (*p* < 0.05). For doxorubicin, which was the positive control, an IC_50_ value of 5.21 ± 0.01 μg/mL was found on the PC-3 cell line (Table 6).

**Table 6:** Cytotoxic Activities of Methanol and Aqueous Extracts of *E. prunastri* against HT-29, PC-3, and A549 Cancer Cell Lines and CCD18-Co Healthy Human Cell Lines (control) as Determined by MTT Assay^a^

	**cancer cell lines**			**human cell lines**
*E. prunastri*	**HT-29**	**PC-3**	**A549**	**nontumorigenic** (CCD18-Co)
	**IC**_**50**_ (μg/mL)			
methanol extract	100* ± 0.04	146* ± 0.05	112* ± 0.06	>200
aqueous extract	>200	>200	>200	>200
cisplatin	4.97* ± 0.01		2.95* ± 0.01	
doxorubucin		5.21* ± 0.01		

a Values are shown as mean ± SD (*n* = 3). * *p* < 0.05.

In agreement with our findings, Yumrutas et al. reported that the methanolic and aqueous extracts of *E. prunastri* were found to possess antiproliferative activities against MCF-7.^13^ Likewise, previous studies reported that the MeOH extract of *E. prunastri* had antiproliferative activities on murine myeloma cells (P3 × 63-Ag8.653) and HCT-116 colon cancer cell lines.^61,98^ Moreover, among different extracts of *E. prunastri*, the methanolic extract exhibited great cytotoxicity on murine Lewis lung carcinoma cells (ATCC CRL-1642) (IC_50_ = 18.7 ± 2.8 μg/mL).^61^ The methanolic extracts of *E. prunastri* have detected the cell viability for human astrocytoma (U373-MG: 19.43% at 10 μg/mL) and human neuroblastoma (SH-SY5Y: 17.32% at 0.5 μg/mL). Queffelec et al. reported that a biopolymer obtained from *E. prunastri* exhibited antiproliferative activity against human lung carcinoma (A549:44 ± 1%), human colon carcinoma (HT-29:37 ± 2%), and human cervix adenocarcinoma (HeLa-229:35 ± 1%). Aqueous extracts from both 140 and 200 °C (microwave-assisted water extraction) treatments showed similar results for A549 (with values of 30 ± 2%) and HeLa-229 (32 ± 5%). The lowest percentages of cellular inhibition were detected for HT-29, with values of 27 ± 2% (200 °C) and 14 ± 1% (140 °C).^24^ Kosanić et al. demonstrated that the Soxhlet treatment with acetone extract of *E. prunastri* showed cytotoxic effects on FemX (human melanoma) and LS174 (human colon carcinoma) cell lines with IC_50_ values of 120.89 ± 1.55 and 120.79 ± 1.01 μg/mL, respectively.^61^

The difference in cytotoxic activity recorded between the two extracts could be explained by their chemical characteristics, the large quantity of some phenolic compounds (rosmarinic acid, caffeic acid, etc.), or the exclusive availability of others (myricetin). Similarly, the cytotoxic activity results from the synergistic or antagonistic effect of several compounds.^12,99,100^ Accordingly, recent reports have proposed that phenolic compounds such as those found in *E. prunastri* may be effective inhibitors of cancer cell proliferation. Compounds such as quercetin and kaempferol exhibited potent cytotoxic and apoptotic effects on HCT-116 cells. In fact, they reduced the oxidative nature of cancer cells and triggered apoptosis by decreasing the expression of the IAP protein family (BIRC2, BIRC3, BIRC4, and BIRC7), which are responsible for cancer progression.^101^ Furthermore, rosmarinic acid was reported to attenuate oxygen-glucose deprivation-induced apoptosis and cytotoxicity, block TNF-α-induced nuclear transcription factor-κB (NF-κB) activation, and decrease high-mobility group box 1 (HMGB1) expression.^102,103^ Ben Sghaier et al. reported the anticarcinogenic role of rutin in suppressing the viability of cancer cells, decreasing the superoxide production, and influencing the adhesion and migration of A549 and HT-29 cells.^104^ Numerous phenolic compounds have anticancer properties and are thought to be promising therapeutic agents. They can induce apoptosis, block the cell cycle, induce DNA damage and death in cancer cells, and regulate multiple cell signaling pathways involved in the progression of cancer. Quercetin, hesperidin, naringin, kaempferol, p-coumaric acid, rutin, ferulic acid, chlorogenic acid, caffeic acid, and gallic acid are a few examples of these compounds.^104,105^ On the other hand, both extracts showed selective cytotoxic effects on cancer cells, with no adverse effects on healthy cells. This leads to the anticancer effect of phenolics since they have the capacity to modulate carcinogen metabolism and gene expression to arrest the cell cycle and induce apoptosis.^106^ Many previous studies have reported the selective cytotoxic effects of phenolics such as chlorogenic acid, caffeic acid, cinnamic acid, p-coumaric acid, apigenin, catechin derivatives, 4-hydroxybenzoic acid, quercetin, hesperidin, myricetin, taxifolin, and naringenin on different cancer cells.^107,108^ Therefore, we believe that the selective cytotoxic effect of both extracts on cancer cells may derive from their phenolic content since *E. prunastri* revealed a range of those compounds herein. *E. prunastri* is commonly consumed as a spice in Algeria. Earlier, it has been demonstrated that cancer cells are more sensitive to the inhibitory effect of spices on DNA, RNA, and protein synthesis than healthy cells.^109,110^ Both *E. punastri* extracts may exert their anticancer effects via inhibition of RNA and DNA synthesis,^111^ apoptosis induction,^112^ and reduction of self-renewal gene expression.^113^ More research is required to clarify the mechanisms underlying the anticancer activity of *E. prunastri* (induction of apoptosis, cell cycle arrest, molecular pathways, etc.). In addition to the antioxidant and various biological activities of rosmarinic acid, caffeic acid, and cinnamic acid, they also have cytotoxic activity against cancer cells such as colon/colorectal, lung, breast, skin, and melanoma.^114−117^ According to investigations, the synergistic effect of phenolic substances including apigenin, caffeic acid, quercetin, and rosmarinic acid enhances cytotoxic activity.^118^ In this research, we believe that the high amounts of rosmarinic acid, quercetin, caffeic acid, and apigenin compounds in the methanol extract may be responsible for the cytotoxic activity of the methanol extract.

These results revealed that the extracts of *E. prunastri* are potent antioxidants owing to their radical scavenging capacity and ability to suppress cancer cell growth. These interactions may result from the presence of molecules with anticancer and antioxidant potential that lead to the induction of apoptotic pathways and selective cytotoxic effects.^119,120^ Recently, the search for new antioxidants with cytotoxic potential is desirable,^121^ and *E. prunastri* demonstrated herein may represent interesting targets for this purpose. Taken together, this report highlights the potential of *E. prunastri* and its phenolic compounds as promising sources of anticancer and antioxidant agents that could lead to drug discovery by maneuvering them as effective and safe drugs. Further studies should be carried out to understand the mechanisms involved in these effects. In addition, insight into the toxicological properties of *E. prunastri* is required, especially since the relationship between its daily consumption as a spice in North Africa and its possible preventive effect may be investigated. Furthermore, in silico research should be conducted to confirm the pharmacokinetic parameters and implicated pathways of *E. prunastri* phenolics, followed by isolation of the best candidates as bioactive compounds. Subsequently, in vivo applications using animal models are required to prove their safety and their therapeutic index before clinical validation.

## 4 Conclusions

The methanolic and aqueous extracts of *E. prunastri* have demonstrated significant antioxidant and anti-inflammatory effects in the current investigation. The cytotoxic activities of both extracts of *E. prunastri* against colon (HT-29), prostate (PC-3), lung (A549) cancer cells, and human healthy (CCD18-Co) cell lines were investigated for the first time. Interestingly, the methanolic extract exerted promising cytotoxic effects against HT-29, PC-3, and A549 cell lines, but not against the nontumor cell lines. These promising and selective effects were attributed to the presence of several phenolic compounds such as rosmarinic acid, caffeic acid, kaempferol, chlorogenic acid, and quercetin. Further research is necessary to completely understand the precise processes by which these substances contribute to the medicinal and health-promoting activities of this lichen. Moreover, to the best of our knowledge, little data are available on *E. prunastri* extracts in the literature. Fortunately, the use of this plant in traditional medicine as a remedy against cancer and inflammatory diseases is scientifically supported by this report. Although there is evidence that indigenous people benefit from consuming *E. prunastri* as a complementary diet during therapies, it has not been demonstrated that regular consumption of this spice is completely safe. Future work will help end the controversy and identify preventive and therapeutic doses. Thus, this lichen requires more in vitro, in silico, and in vivo investigations, including isolation and characterization of the components linked to these bioactivities.^66,69,103^
